# 糖酵解重编程与乳酸化串扰在肿瘤中的研究进展

**DOI:** 10.3779/j.issn.1009-3419.2026.101.02

**Published:** 2026-01-20

**Authors:** Yilin FENG, Ying SUN, Xu HAO, Huitong YANG, Anqi LU, Yuan LIU, Jinchan XIA, Long FENG, Min JIANG, Xiru ZHAO

**Affiliations:** 450046 郑州，河南中医药大学医学院; Medical College of Henan University of Chinese Medicine, Zhengzhou 450046, China

**Keywords:** 乳酸化修饰, 糖酵解重编程, 肿瘤微环境, 免疫逃逸, 靶向治疗, Lactylation, Glycolytic reprogramming, Tumor microenvironment, Immune evasion, Targeted therapy

## Abstract

代谢重编程是癌症的核心特征，其中Warburg效应驱动的有氧糖酵解导致肿瘤微环境中乳酸大量积累。长期以来，乳酸被视为代谢终产物，然而近年研究发现，它作为一种重要的信号分子，通过诱导新型翻译后修饰——乳酸化修饰，深刻影响肿瘤进程。乳酸化修饰由乳酸驱动，发生在组蛋白与非组蛋白上，受“writer”“eraser”和“reader”机制的精密调控，从而改变靶蛋白功能与基因表达。本综述系统探讨了糖酵解重编程与乳酸化之间存在的双向调控网络：一方面，糖酵解关键调控因子促进乳酸生成，进而提升乳酸化水平；另一方面，乳酸化又能反馈调节糖酵解关键酶的活性与表达，形成促癌正反馈循环。这一互作在肿瘤增殖、转移、DNA损伤修复及免疫逃逸中发挥核心作用。基于此，靶向乳酸生成、乳酸转运或乳酸化修饰过程本身，已成为极具潜力的抗肿瘤策略，并与免疫检查点抑制剂等现有疗法展现出协同前景。深入解析糖酵解-乳酸化轴将为开发新型肿瘤治疗手段提供重要理论基础。

## Abstract (en)

Metabolic reprogramming is a hallmark of cancer, with the Warburg effect-driven aerobic glycolysis leading to a substantial accumulation of lactate in the tumor microenvironment. For a long time, lactate was considered a mere metabolic end product; however, recent studies have found that it acts as an important signaling molecule, profoundly influencing tumor progression by inducing a novel post-translational modification - lactylation. Lactylation, driven by lactate, occurs on both histones and non-histone proteins and is finely regulated by the 'writer' 'eraser' and 'reader' mechanisms, thereby altering the function of target proteins and gene expression. This review systematically explores the bidirectional regulatory network between glycolytic reprogramming and lactylation: on one hand, key glycolytic regulators promote lactate production, thereby increasing lactylation levels; on the other hand, lactylation can feedback to regulate the activity and expression of key glycolytic enzymes, forming a pro-tumor positive feedback loop. This interaction plays a central role in tumor proliferation, metastasis, DNA damage repair, and immune evasion. Consequently, targeting lactate production, lactate transport, or the lactylation process itself has emerged as a highly promising anti-cancer strategy and shows potential synergy with existing therapies such as immune checkpoint inhibitors. In-depth analysis of the glycolysis-lactylation axis will provide a crucial theoretical basis for developing novel cancer treatment approaches.

代谢重编程是癌症的重要特征之一，表现为肿瘤细胞倾向于依赖糖酵解而非氧化磷酸化来获取能量^[1]^。20世纪初，Warburg^[2]^发现即使在氧气充足的条件下，癌细胞仍大量进行糖酵解并产生乳酸，该现象后被命名为Warburg效应。近年研究^[3]^表明，乳酸不仅是一种能量代谢产物，还具有广泛的信号调控功能。它可在肿瘤微环境（tumor microenvironment, TME）中积累，并通过酸化环境促进血管生成、肿瘤转移、耐药性及免疫逃逸等恶性进程。此外，乳酸还可作为信号分子调控免疫细胞功能、代谢重编程及免疫监视机制，深刻影响肿瘤进展^[4]^。近年来，乳酸化修饰（lysine lactylation, Kla）的发现进一步拓展了乳酸的功能范畴，揭示其在基因表达调控及细胞信号转导中的重要作用。

乳酸化是一种新发现的翻译后修饰（post-translational modification, PTM），代表了一类新兴的表观遗传调控机制，可参与调控蛋白质功能和疾病发生^[5]^。该修饰可发生在组蛋白与非组蛋白上，不仅参与多种生理过程，还在包括癌症在内的多种疾病的发生与发展中扮演关键角色^[6]^。在TME中，肿瘤细胞、肿瘤干细胞以及肿瘤浸润免疫细胞中的Kla，可通过影响组蛋白表观遗传状态或改变非组蛋白的结构功能，进而调控下游基因表达及信号通路。因此，靶向蛋白质乳酸化已成为癌症治疗中一个具有潜力的研究方向。尽管已有研究对乳酸诱导的乳酸化进行了初步探索，但目前仍缺乏将其与糖酵解重编程系统联系并基于该关联开展肿瘤干预策略的研究。本文旨在系统梳理糖酵解重编程与乳酸化之间的互作关系，阐述Kla的分子调控机制，并总结针对该通路的抗肿瘤研究进展，以期为开发靶向乳酸化的新型肿瘤治疗策略提供理论依据。

## 1 乳酸与乳酸化

Zhang等^[7]^研究人员通过质谱分析发现，组蛋白水解肽中赖氨酸残基上存在与乳酸基修饰一致的质量位移，提示可能存在乳酸辅酶A（lactoyl-CoA）的生成，为Kla提供了早期实验证据。该团队首次在组蛋白中鉴定出赖氨酸Kla。随着研究的深入，更多组蛋白Kla位点被陆续鉴定和验证。其中，H3K9la、H3K18la、H4K5la和H4K12la等被证实对肿瘤发生、进展及免疫逃逸具有重要调控作用。Kla不局限于组蛋白，也广泛存在于非组蛋白中，显著扩展了其功能多样性。非组蛋白的Kla可能影响其酶活性、亚细胞定位及蛋白-蛋白相互作用，进而通过多种机制调控下游基因表达及信号传导。Kla范围的不断拓展，凸显了其在蛋白质功能调控中的广泛潜力。从分子机制层面看，这种新型修饰的生物学效应实现，依赖于一个高度协调的代谢-表观遗传调控网络。目前普遍认为，Kla的核心调控过程主要包括3个环节：乳酸的生成与积累、乳酸的跨膜运输，以及依赖于“writer”“reader”“eraser”的lactoyl-CoA介导的修饰循环。

## 2 糖酵解重编程与乳酸化

糖酵解重编程与Kla之间存在着复杂的双向调控关系，共同参与肿瘤的发生与发展进程。一方面，Kla水平通常与糖酵解重编程存在一定的相关性；另一方面，Kla可通过影响糖酵解关键酶的转录活性、结构稳定性和功能状态，反向调节糖酵解通路的活性。

### 2.1 糖酵解重编程诱导乳酸化

糖酵解重编程可以通过多种机制影响Kla，其中缺氧诱导因子α（hypoxia-inducible factor-α, HIF-α）、原癌基因c-Myc以及腺苷酸激活蛋白激酶（AMP-activated protein kinase, AMPK）等关键因子在此过程中发挥重要调节作用。HIF-1α作为缺氧应答的核心转录因子，不仅可激活多种缺氧响应基因，还能够通过调控糖代谢重编程促进糖酵解、抑制三羧酸循环，从而增加乳酸生成。其机制在于HIF-1α与靶基因启动子区的缺氧反应元件（hypoxia-responsive element, HRE）结合，直接上调包括葡萄糖转运蛋白1（glucose transporter 1, GLUT1）、己糖激酶（hexokinase, HK）、乳酸脱氢酶（lactate dehydrogenase, LDH）、丙酮酸激酶M2型（pyruvate kinase M2, PKM2）、磷酸果糖激酶（phosphofructokinase, PFK）在内的多个糖酵解关键基因的转录，促进糖酵解^[8,9]^。与HIF-α类似，c-Myc直接参与调控糖酵解相关基因的转录，增强有氧糖酵解进程^[10]^。例如，c-Myc的过表达能够促进前列腺癌（prostatic carcinoma, PCa）细胞的糖酵解和乳酸产生，上调PCa的泛乳酸化并发生自身Kla^[11]^。同时，HIF-α和c-Myc在调控乳酸化过程中还存在协同作用。例如在胰腺导管腺癌（pancreatic ductal adenocarcinoma, PDA）中，核仁和纺锤体相关蛋白1（nucleolar and spindle-associated protein 1, NUSAP1）可与HIF-α、c-Myc在LDHA启动子区形成转录复合体，共同增强LDHA的表达，促进糖酵解并提高NUSAP1乳酸化水平，从而抑制NUSAP1的蛋白降解^[12]^。而NUSAP1是一种参与肿瘤生物学的微管相关蛋白，与肿瘤不良预后有关。此外，AMPK通常被认为是细胞能量传感器和糖酵解的负调控因子，可抑制Warburg效应^[13]^。然而，在未分化间变性甲状腺癌（anaplastic thyroid cancer, ATC）中，AMPK通路激活反而增加了糖酵解通量，提高了整体泛乳酸化及组蛋白H4K12乳酸化水平。

综上所述，缺氧微环境及癌基因激活等多种因素，可通过促进有氧糖酵解显著调节Kla水平。

### 2.2 乳酸化对糖酵解重编程的调节

Kla以糖酵解代谢产物乳酸为底物，通过调控代谢相关酶的表达及活性，充当糖酵解的反馈调节剂^[14]^。目前普遍认为，乳酸化对糖酵解代谢酶的调控主要体现在转录水平和结构功能两个层面。HK是葡萄糖代谢中的关键酶，催化葡萄糖磷酸化为葡萄糖-6-磷酸，该反应是糖酵解的第一个不可逆步骤^[15]^。HK存在多个亚型，如HK1、HK2、HK3和HK4^[16]^。其中，HK1和HK2的表达和功能在癌症中受到组蛋白Kla的调控^[17]^。在非小细胞肺癌（non-small cell lung cancer, NSCLC）细胞中，乳酸处理可下调HK1和PKM等糖酵解关键酶的基因表达，同时上调琥珀酸脱氢酶（succinate dehydrogenase, SDH）和异柠檬酸脱氢酶3γ（isocitrate dehydrogenase 3 gamma, IDH3G）等三羧酸循环相关酶的转录水平，有助于维持线粒体稳态。机制研究^[17]^表明，HK1和IDH3G启动子区域存在丰富的组蛋白Kla，提示乳酸化可能通过表观遗传机制参与代谢重编程的调控。PKM2是一种糖酵解限速酶，可以以单体、二聚体及四聚体形式存在。随着PKM2的活性降低，其单体及二聚体可以易位到细胞核，通过与HIF-1α相互作用上调多种糖酵解相关酶的表达，促进糖酵解。Wang等^[18]^发现，在促炎型巨噬细胞中，乳酸可诱导PKM2第26位赖氨酸发生乳酸化，增强PK活性并抑制其由四聚体向二聚体的构象转变，减少核转位，这一修饰抑制了糖酵解重编程。此外，在胰腺癌中，H3组蛋白K18位发生Kla产生H3K181a，可增强苏氨酸酪氨酸激酶（threonine tyrosine kinase, TTK）和有丝分裂检查点丝氨酸/苏氨酸激酶B（BUB1 mitotic checkpoint serine/threonine kinase B, BBUB1B）的转录，而TTK和BUB1B又可上调Kla相关“writer”即P300/CBP的转录。同时，TTK还能够激活糖酵解相关酶LDHA，刺激乳酸的生成，进一步提高组蛋白Kla水平。这建立了涉及H3K181a和TTK/BUB1B的糖酵解正反馈循环，永久刺激糖酵解和肿瘤增殖^[19]^。此外，在HCC中的富集分析^[20]^也显示，Kla显著影响代谢酶、氧化还原酶及脱氢酶的活性，进一步表明Kla在糖酵解重编程中的重要作用。

因此，Kla与糖酵解重编程形成正反馈回路，肿瘤细胞通过糖酵解重编程产生大量乳酸，进而触发蛋白质Kla。反之，Kla又进一步促进肿瘤细胞的糖酵解重编程。

## 3 糖酵解重编程与乳酸化的双向调控在肿瘤发展中的作用

糖酵解重编程与乳酸化之间的双向调控作用在肿瘤的发生发展中发挥重要作用。一方面，糖酵解的增强导致乳酸大量积累，后者作为关键底物，在特定酶的作用下可催化组蛋白及非组蛋白进行Kla，直接影响细胞的表观遗传状态并激活致癌基因转录；另一方面，Kla还能重塑TME，通过调控免疫细胞功能促进免疫抑制，从而协同驱动肿瘤恶性进展。

### 3.1 糖酵解调控乳酸化发挥促肿瘤作用

正如前文所述，糖酵解重编程通过调控乳酸的产生与积累影响乳酸化。近年来，多项研究揭示了乳酸化在不同肿瘤类型中的核心作用，包括眼黑色素瘤、ATC、透明细胞肾细胞癌（clear cell renal cell carcinoma, ccRCC）^[21]^、结直肠癌、胃癌（gastric carcinoma, GC）、急性髓性白血病（acute myeloid leukemia, AML）、膀胱癌、NSCLC^[17]^、肝细胞癌^[20]^、胶质瘤^[22]^、PCa^[23]^和PDA^[24]^等。其分子机制主要涉及组蛋白与非组蛋白两大类修饰底物，通过调控肿瘤相关基因表达、促进肿瘤细胞增殖与迁移、诱导肿瘤细胞耐药等途径驱动肿瘤恶性进展^[25]^。

#### 3.1.1 组蛋白乳酸化的促癌机制

H3K18作为一个经典的组蛋白Kla位点，在多种肿瘤中发挥转录激活功能。在眼黑色素瘤中，H3K18乳酸化水平升高可促进YTH N6-甲基腺苷RNA结合蛋白F2（YTH N6-methyladenosine RNA binding protein F2, YTHDF2）表达。YTHDF2作为一种m^6^A阅读蛋白能够特异性识别并降解包括周期昼夜节律调节因子1（period circadian regulator 1, PER1）和p53在内的多个抑癌基因的m^6^A修饰转录本，加速眼部黑色素瘤的发生^[24,26]^。此外，在结肠癌（colorectal cancer, CRC）中，H3K18乳酸化可促进METTL3的表达上调，通过m^6^A修饰增强Janus激酶1（Janus kinase 1, JAK1）的翻译效率，并激活信号转导与转录激活因子3（signal transducer and activator of transcription 3, STAT3），促进JAK1发生磷酸化，加速肿瘤进展^[27]^。另有研究^[28]^显示，H3K18乳酸化还可调控自噬调节因子（rubicon like autophagy enhancer, Rubcnl）的转录，促进结直肠癌细胞的增殖与存活。Hou等^[29]^在乳腺癌中的研究表明，钾双孔结构域通道亚家族K成员1（potassium two pore domain channel subfamily K member 1, KCNK1）通过激活LDHA调控H3K18乳酸化，推动癌细胞增殖。在ccRCC中，抑癌蛋白VHL（von Hippel-Lindau）可诱发组蛋白泛乳酸化及H3K18乳酸化，促进血小板衍生生长因子受体β（platelet derived growth factor receptor beta, PDGFRβ）的转录，加速肿瘤进展^[21]^。在膀胱癌中，H3K18乳酸化通过Hippo信号通路促进肿瘤发生发展，该过程可被糖酵解负调控因子circXRN2所抑制^[30]^。

除H3K18外，其他组蛋白乳酸化位点也具有重要功能。血管内皮生长因子（vascular endothelial growth factor, VEGF）信号通路激活可诱导内皮细胞H3K9乳酸化，该修饰通过特异性结合于促血管生成相关基因的启动子区域，增强其转录活性，促进肿瘤侵袭与转移^[31]^。在肝癌中，H3K9和H3K56乳酸化通过增强CD133、Cyclin D1/E1等基因表达，促进肝癌干细胞（liver cancer stem cells, LCSCs）的自我更新和细胞周期进程，基因编辑敲除这些修饰位点可显著减弱肿瘤干细胞的致瘤性^[32]^。在ATC中，甲状腺癌驱动因子BRAF V600E发生突变。它通过增强糖酵解活性提高H4K12乳酸化水平，激活增殖相关基因，促进细胞增殖并抑制凋亡^[33]^。此外，H4K12乳酸化还可以激活细胞周期蛋白B1（cyclin B1, CCNB1）基因表达，影响NSCLC的化疗耐药性^[34]^。

#### 3.1.2 非组蛋白乳酸化的促癌机制

非组蛋白乳酸化在肿瘤进展中同样发挥关键作用。研究^[35]^发现在宫颈癌中，致癌蛋白DCBLD1（discoidin, CUB and LCCL domain containing 1）在K172位点发生乳酸化，激活磷酸戊糖途径，为核酸合成提供前体，驱动肿瘤细胞的增殖与转移。在GC中，铜胁迫可增强甲基转移酶样蛋白16（methyltransferase-like proteins 16, METTL16）K229位点的乳酸化，该修饰通过促进铁氧还蛋白1（recombinant ferredoxin 1, FDX1）mRNA发生m^6^A修饰以增强其稳定性，进而促进铜依赖性GC细胞凋亡^[36]^。此外，在胰腺癌中，Ras同源家族成员F（Ras homolog family member F, RHOF）激活PKM2介导的糖酵解通路，促进蜗牛家族转录抑制因子1（snail family transcriptional repressor 1, Snail1）发生Kla并易位至细胞核，增强肿瘤细胞的内皮-间质转化能力，驱动其迁移与侵袭^[37]^。在PCa中，乳酸转运体MCT1介导的乳酸内流上调HIF-1α的乳酸化水平并稳定其表达，增强细胞迁移诱导蛋白（cell migration inducing hyaluronidase 1, KIAA1199）的转录，促进血管生成和肿瘤转移^[23]^。

#### 3.1.3 乳酸化介导的肿瘤耐药机制

糖酵解重编程和Kla还可介导肿瘤细胞对化疗和靶向药物的耐药性。在结直肠癌干细胞中，组蛋白H4K12乳酸化通过转录激活谷氨酸-半胱氨酸连接酶（glutamate-cysteine ligase catalytic subunit, GCLC），抑制铁死亡，导致化疗耐药；敲除Kla的关键催化因子p300或糖酵解限速酶，可有效降低H4K12乳酸化水平，并显著增强肿瘤细胞对化疗药物的敏感性^[38]^。此外，基于结直肠癌患者来源的类器官及异种移植模型（patient-derived xenograft, PDX）的研究^[39]^证实，Warburg效应增强可提升肿瘤细胞的同源重组（homologous recombination, HR）修复能力，诱导其对化疗药物产生耐药性。

综上所述，Kla作为糖酵解重编程的关键功能输出，在肿瘤增殖、迁移及耐药中扮演重要角色。然而，乳酸化在不同肿瘤类型中的特异性功能及调控网络仍存在大量未知，其精确的分子机制和临床转化潜力亟待进一步深入研究。

### 3.2 乳酸化调控糖酵解发挥免疫逃逸作用

TME是一个高度复杂且异质性的生态系统，其代谢特征深刻影响着免疫应答的效力^[40]^。糖酵解重编程在塑造免疫抑制性TME中扮演关键角色^[41]^，其终产物乳酸不仅是能量代谢的载体，更作为Kla的前体，通过修饰组蛋白及非组蛋白，广泛调控各类免疫细胞的功能状态，从而促进肿瘤免疫逃逸^[42,43]^。

#### 3.2.1 乳酸化对髓系免疫细胞的调控

巨噬细胞作为TME中的关键免疫群体，其功能极化受到Kla的精细调控。在M1型巨噬细胞极化早期，糖酵解途径的激活促使乳酸积累。随着极化进程推进，细胞内乳酸水平显著上升，诱导组蛋白乳酸化水平增加^[44]^。研究^[45]^表明，H3K18乳酸化可促进M2型巨噬细胞相关基因的转录，从而驱动促炎型M1巨噬细胞向抗炎型M2巨噬细胞转化，重塑肿瘤免疫微环境，抑制抗肿瘤免疫应答，加速肿瘤的生长与转移进程。此外，Cai等^[46]^研究发现，在肝癌中，富丝氨酸和精氨酸的剪接因子10（serine and arginine rich splicing factor 10, SRSF10）通过结合MYB基因的3' UTR区域，增强关键糖酵解酶GLUT1、HK1和LDHA mRNA的稳定性，进而上调其表达，导致细胞内及微环境中乳酸水平升高。积累的乳酸进一步诱导H3K18乳酸化，促进SRSF10的表达，形成SRSF10-糖酵解-H3K18乳酸化之间的正反馈循环。该循环通过促进M2型巨噬细胞极化，抑制CD8^+ ^T细胞在TME中的浸润及其干扰素γ（interferon-gamma, IFN-γ）的分泌，共同塑造免疫抑制微环境。肿瘤浸润性髓样细胞（tumor-infiltrating myeloid cells, TIMs）作为另一类免疫抑制亚群，其功能同样受到乳酸化调控。H3K18乳酸化可促进TIMs中甲基转移酶METTL3的表达，并使METTL3的锌指结构域发生乳酸化，促进JAK1的mRNA甲基化修饰，结合YTHDF1提高翻译效率，增强JAK1/STAT3信号通路的激活，进而强化TIMs的免疫抑制功能，最终促进肿瘤免疫逃逸^[27]^。

#### 3.2.2 乳酸化对淋巴系免疫细胞的调控

肿瘤浸润淋巴细胞（tumor infiltrating lymphocytes, TILs）是一种存在于TME中的免疫细胞，可以直接或间接参与免疫反应，影响肿瘤的生长。CD8^+^ T细胞作为抗肿瘤免疫的核心效应细胞，其活化与功能同样受到组蛋白Kla的影响。研究^[47]^表明，H3K18和H3K9位点的乳酸化水平变化可显著影响CD8^+^ T细胞的抗肿瘤免疫反应。在AML中，STAT5上调糖酵解导致乳酸积累，通过促进H4K5乳酸化增强免疫检查点蛋白程序性死亡配体-1（programmed cell death-ligand 1, PD-L1）的转录^[48]^，进而诱导CD8^+ ^T细胞凋亡，促进肿瘤免疫逃逸。值得注意的是，乳酸化还直接参与辅助性T17细胞（T helper cell 17, Th17）的分化调控。转录因子IKAROS家族锌指蛋白1（IKAROS family zinc finger protein 1, Ikzf1）第164位赖氨酸的Kla，可以调控Th17分化相关基因如RUNT相关转录因子1（runt-related transcription factor 1, Runx1）、Toll样受体4（Toll-like receptor 4, TLR-4）、白细胞介素（interleukin, IL）-2和IL-4的表达，增强Th17细胞的分化，塑造免疫抑制性微环境，为肿瘤生长提供庇护^[49]^。此外，调节性T细胞（regulatory T cells, Tregs）是维持免疫耐受并促进肿瘤免疫逃逸的关键细胞亚群，Kla可影响Tregs的增殖与分化。研究^[50]^发现，肿瘤组织中高表达的载脂蛋白C2（apolipoprotein C2, APOC2）在乳酸刺激下发生K70位点乳酸化，从而促进游离脂肪酸（free fatty acids, FFA）释放，并驱动Tregs扩增。膜组织延伸刺突蛋白（membrane-organizing extension spike protein, MOESIN）第72位赖氨酸的Kla可增强其与转化生长因子-β（transforming growth factor-β, TGF-β）受体间的相互作用，进而激活下游SMAD同源物3（recombinant SMAD family member 3, SMAD3）信号通路，促进Tregs的生成^[51]^，影响肿瘤发生。此外，视黄酸诱导基因蛋白I（retinoic acid-inducible gene-I, RIG-I）的Kla可抑制巨噬细胞中核因子-κB向NOD样受体热蛋白结构域相关蛋白3（NOD-like receptor thermal protein domain associated protein 3, Nlrp3）启动子的募集，导致Nlrp3转录水平下降，削弱Tregs的免疫抑制功能及CD8^+^ T细胞的抗肿瘤活性^[52]^，促进肿瘤恶性进展。

综上所述，Kla通过调控髓系和淋巴系多个免疫细胞亚群的功能状态，在糖酵解驱动的肿瘤免疫逃逸中发挥核心作用。

## 4 糖酵解重编程调控乳酸化在肿瘤免疫治疗中的应用

Kla通过特异性作用于组蛋白和非组蛋白的特定位点，调控下游基因的转录活性及蛋白质功能，并借助介导TME中的免疫抑制与代谢重编程过程，促进肿瘤的发生与恶性进展。基于以上机制，靶向乳酸化及其背后的糖酵解重编程已成为癌症治疗的新兴研究方向。目前主要治疗策略包括3个方面：一是抑制乳酸生成及其细胞外转运，从而降低TME中乳酸的积累，削弱Kla水平，以缓解免疫抑制状态；二是开发可直接作用于Kla过程的小分子抑制剂，阻断其对肿瘤细胞及免疫细胞功能的异常调控；三是将乳酸化靶向干预与肿瘤免疫治疗相结合，通过协同作用提升抗肿瘤疗效。

### 4.1 调控糖酵解抑制乳酸化

乳酸的产生与转运是Kla发生的重要促进因素。而糖酵解作为乳酸生成的核心代谢通路，其重编程过程被视为影响Kla的关键调控环节。LDH是糖酵解途径中的关键酶，负责催化该通路的终末步骤，即将丙酮酸转化为乳酸^[53]^。LDH是由LDHA和LDHB两种亚基共同构成的四聚体蛋白^[54]^。研究^[55]^表明，在LDHA被沉默后，高迁移率族蛋白B1（high mobility group box 1, HMGB1）启动子区域的Kla富集显著减少。为深入验证LDHA在Kla中的关键作用，研究人员进一步在人肾癌细胞系786-O和A498中敲低LDHA表达，均观察到整体乳酸化水平显著下降。上述结果一致表明，靶向LDHA可能成为调控Kla的有效策略。研究^[56]^表明，草酰胺（Oxamate）和二氯乙酸（Dichloroacetate, DCA）作为LDH的抑制剂，可有效抑制糖酵解并降低细胞内乳酸水平，进而抑制H3K18乳酸化，最终下调与肝星状细胞活化相关基因的表达。目前有关DCA的临床II期试验（NCT05120284）正在进行，这将为进一步评估其在更广泛癌症中的疗效及治疗潜力提供依据。另有研究^[57]^指出，将LDHA抑制剂与糖酵解关键调控酶丙酮酸脱氢酶激酶1（pyruvate dehydrogenase kinase 1, PDK1）的抑制剂联合使用，可在肺腺癌模型中产生协同抑制肿瘤生长的效应。因此，以LDH为靶点的抑制剂可能代表了一类通过干预Kla进而发挥抗肿瘤作用的潜在治疗策略。

此外，药理学层面对糖酵解的抑制也被证实可显著降低组蛋白乳酸化水平。研究^[58,59]^表明，使用2-DG处理能够通过抑制HK显著减少H3K9与H3K18的乳酸化。环状RNA circXRN2作为糖酵解和乳酸生成的负调控因子，可通过结合大肿瘤抑制激酶1（large tumor suppressor kinase 1, LATS1），保护其免受斑点型锌指结构蛋白（speckle-type poz protein, SPOP）介导的泛素化降解，从而激活Hippo信号通路，最终抑制H3K18乳酸化所驱动的膀胱癌进展^[30]^。值得关注的是，靶向糖酵解-乳酸化轴在多种癌症类型中均显示出治疗潜力。在NSCLC中，源自木兰植物的活性成分辛夷脂素（Fargesin, FGS）可通过靶向PKM2抑制有氧糖酵解及其下游H3组蛋白乳酸化相关信号转导，展现出抗肿瘤效应^[60]^。此外，α-氰基-4-羟基肉桂酸（α-cyano-4-hydroxycinnamic acid, CHCA）作为MCT1选择性抑制剂，可显著抑制胶质母细胞瘤（glioblastoma multiforme, GBM）细胞的增殖、迁移和存活^[61]^。MCT1/MCT2双重抑制剂AZD3965^[62]^已进入针对晚期癌症患者的I期临床试验阶段（NCT01791595）。目前的研究^[63]^结果显示，其在治疗过程中患者普遍耐受良好，但部分出现限制性毒性，涉及心脏肌钙蛋白升高及视网膜电图改变，这可能与MCT1在视网膜及心肌等正常组织中的广泛表达密切相关。因此，如何优化现有抑制剂的设计，开发具有更高组织选择性和安全性的糖酵解相关酶抑制剂，已成为该领域未来转化研究的重点方向。

### 4.2 调控Kla相关酶抑制乳酸化

近年来，靶向Kla过程中的关键调控因子“writer”“reader”“eraser”，在癌症治疗中引起越来越多的关注^[64]^。以组蛋白乙酰转移酶P300为例，该蛋白被证实可作为Kla的“writer”参与调控。Deng等^[65]^研究发现，在人结肠癌细胞系HCT116中敲低P300能够显著降低乳酸化水平，并有效抑制肿瘤细胞的恶性进展，提示P300可能是乳酸化调控的潜在靶点。然而需注意的是，P300在Kla中并非发挥单一作用。研究^[66]^发现，巨噬细胞通过MCT摄取细胞外乳酸后，除直接通过P300/CBP促进HMGB1乳酸化外，也会被β-arrestin2介导的P300/CBP通过G蛋白偶联受体81（G protein-coupled receptor 81, GPR81）募集至细胞核，刺激HMGB1乙酰化。相反，I类HDAC抑制剂可通过抑制Kla而促进H3K18的乙酰化修饰。这些证据表明乳酸化与乙酰化之间可能存在相互调控的复杂关系。因此，在选择乳酸化特异性干预靶点时，需审慎评估其对乙酰化等修饰途径的潜在影响。此外，在胃癌发生发展中，乳酸与铜离子浓度的共同升高可诱导METTL16蛋白K229位点发生乳酸化，该修饰能够增强伊利司莫（Elesclomol）所诱导的铜离子依赖性细胞凋亡。然而，当使用SIRT2（乳酸化“eraser”）对该位点进行去乳酸化处理后，显著抑制了癌细胞死亡^[67]^。值得注意的是，在此基础上联合应用Elesclomol与AGK2（SIRT2抑制剂），在体外细胞实验和体内动物模型中均能够有效促进铜介导的胃癌细胞死亡，显示出这一协同策略在胃癌治疗中的转化潜力。然而，目前组蛋白乳酸化特定的“writer”“eraser”和“reader”较为匮乏，使其特异性调控变得困难。开发针对乳酸化的特异性抑制剂，进一步探索其乳酸化的机制，还需要更多的研究。

### 4.3 靶向Kla与肿瘤免疫治疗的协同策略

癌症的发生与发展是一个多步骤、多机制参与的复杂病理过程。尽管针对Kla的单一干预具有一定的抗肿瘤潜力，但其单独应用往往难以取得理想的治疗效果。因此，将乳酸化靶向策略与现有免疫治疗方法相结合，已成为拓展其临床转化前景的重要方向。草胺酸盐（Oxamate）和嵌合抗原受体T细胞（chimeric antigen receptor T cell, CAR-T）疗法的联合应用，能够有效降低肿瘤细胞中H3K18乳酸化，抑制乳酸生成，并下调免疫调节基因膜外三磷酸腺苷二磷酸水解酶-1（ectonucleoside triphosphate diphosphohydrolase-1, CD39）、胞外-5'-核苷酸酶（cluster of differentiation 73, CD73）和C-C基序趋化因子受体8（C-C motif chemokine receptor 8, CCR8）启动子区域的活性。这一系列分子变化共同作用，有助于逆转免疫抑制性TME，增强T细胞介导的抗肿瘤免疫应答，从而提升整体治疗效果。

除CAR-T联合策略外，靶向乳酸化在与其他免疫治疗方式协同中也展现出广泛潜力。在贝伐珠单抗耐药的临床前模型^[64]^中，2-DG与贝伐珠单抗联合治疗表现出显著的协同抗肿瘤效果，提示通过抑制组蛋白乳酸化可能增强结直肠癌靶向治疗的临床响应。进一步研究显示，Kla在免疫检查点抑制剂疗效调控中亦扮演关键角色。在一项关于MOESIN蛋白乳酸化的研究中发现，将LDH抑制剂与抗程序性死亡受体1（programmed cell death protein 1, PD-1）联合应用，相较于单用免疫检查点阻断，可更有效降低TME中的乳酸浓度，并显著增强抗肿瘤效应。

此外，研究^[68]^还提示Kla在调控肿瘤治疗耐药性方面具有重要作用。在NSCLC耐药细胞中，组蛋白去乙酰化酶（histone deacetylase 1, HDAC1）表达上调，导致H4K12乳酸化水平下降，染色质可及性降低，削弱了转录因子CCAAT增强子结合蛋白β（CCAAT enhancer binding protein beta, CEBPB）与其下游靶基因醛酮还原酶家族1成员C2（aldo-keto reductase family 1 member c2, AKR1C2）启动子的结合，最终抑制AKR1C2的表达，激活了致癌的哺乳动物雷帕霉素靶蛋白（mammalian target of rapamycin, mTOR）信号通路，驱动耐药表型。在转移性结直肠癌模型中，MRE11的Kla会抑制其参与HR及DNA断裂修复的能力，导致肿瘤细胞对顺铂产生化疗耐药。然而，通过使用细胞穿透肽特异性阻断MRE11乳酸化，可恢复HR功能、逆转耐药性，并增强癌细胞对顺铂及聚ADP核糖聚合酶（poly ADP-ribose polymerase, PARP）抑制剂的敏感性^[39]^。

## 5 小结与展望

Kla与肿瘤细胞的代谢重编程密切相关，在调控细胞功能中发挥着重要作用。相较于传统PTM，乳酸化的动态变化更直接受到营养状态、氧气供应及能量需求的调节，体现出其独特的微环境响应特性。尽管目前对乳酸化的研究已取得显著进展，但仍存在诸多关键问题亟待深入探索。第一，开发TME特异性的乳酸化抑制剂。利用酸性pH或高乳酸浓度作为药物激活开关，提高治疗特异性。同时，系统评估乳酸化抑制剂与现有化疗、免疫治疗的协同效应，特别是在糖酵解高度活跃的耐药肿瘤中，探索基于乳酸化水平的联合治疗新策略。第二，未来研究拓展多个前沿交叉领域，探索肠道菌群代谢产物对全身及肿瘤局部乳酸水平的影响及其对Kla的间接调控作用。第三，建立Kla的临床转化应用体系。将乳酸化谱纳入生物标志物开发，用于预测治疗反应。通过蛋白质组学等技术筛选可在血液、组织等样本中稳定检测的Kla标志物，开发用于肿瘤早期预警、疗效监测和预后判断的诊断试剂盒。同时，发展无创或微创的乳酸化动态监测技术，如开发特异性识别Kla的分子探针，结合影像学手段实现肿瘤代谢状态的实时可视化评估，将为个体化治疗提供重要依据。第四，技术创新驱动研究范式的革新。充分整合与发展前沿技术，利用人工智能与机器学习分析多组学数据，以预测Kla位点及其调控网络。在此基础上，结合单细胞测序及空间转录组学，共同揭示乳酸化在肿瘤异质性和治疗抵抗细胞亚群中的分布，更精准地理解其在肿瘤发生发展过程中的角色。

综上所述，乳酸化在连接糖酵解代谢与肿瘤免疫调控中居于核心地位。深化对“糖酵解-乳酸化”相互作用复杂性的理解，将不仅促进对肿瘤发生发展机制的深入理解，也有望为探索新的治疗靶点以及改善癌症患者的预后提供理论支撑。
